# Dorsal cortex line is more reliable than transepicondylar axis for rotation in revision total knee arthroplasty with severe bone loss

**DOI:** 10.1302/2633-1462.512.BJO-2024-0140.R1

**Published:** 2024-12-02

**Authors:** Mikhail Salzmann, Ellen Kropp, Robert Prill, Nikolai Ramadanov, Marco Adriani, Roland Becker

**Affiliations:** 1 Center or Orthopaedics and Traumatology, University Hospital Brandenburg/Havel, Brandenburg Medical School Theodor Fontane, Brandenburg, Germany; 2 Faculty of Health Sciences Brandenburg, Brandenburg Medical School Theodor Fontane, Brandenburg, Germany; 3 Department of Medical and Surgical Specialties, Radiological Sciences, and Public Health, University of Brescia, Brescia, Italy

**Keywords:** Revision total knee arthroplasty, Revision TKA, Anatomical landmarks in revision TKA, Dorsal cortex line, DCL, transepicondylar axis, bone loss, revision total knee arthroplasty, CT scans, distal femur, femoral component, femoral component rotation, total knee arthroplasty (TKA), intraclass correlation coefficient (ICC), femoral condyles

## Abstract

**Aims:**

The transepicondylar axis is a well-established reference for the determination of femoral component rotation in total knee arthroplasty (TKA). However, when severe bone loss is present in the femoral condyles, rotational alignment can be more complicated. There is a lack of validated landmarks in the supracondylar region of the distal femur. Therefore, the aim of this study was to analyze the correlation between the surgical transepicondylar axis (sTEA) and the suggested dorsal cortex line (DCL) in the coronal plane and the inter- and intraobserver reliability of its CT scan measurement.

**Methods:**

A total of 75 randomly selected CT scans were measured by three experienced surgeons independently. The DCL was defined in the coronal plane as a tangent to the dorsal femoral cortex located 75 mm above the joint line in the frontal plane. The difference between sTEA and DCL was calculated. Descriptive statistics and angulation correlations were generated for the sTEA and DCL, as well as for the distribution of measurement error for intra- and inter-rater reliability.

**Results:**

The external rotation of the DCL to the sTEA was a mean of 9.47° (SD 3.06°), and a median of 9.2° (IQR 7.45° to 11.60°), with a minimum value of 1.7° and maximum of 16.3°. The measurements of the DCL demonstrated very good to excellent test-retest and inter-rater reliability coefficients (intraclass correlation coefficient 0.80 to 0.99).

**Conclusion:**

This study reveals a correlation between the sTEA and the DCL. Overall, 10° of external rotation of the dorsal femoral cortical bone to the sTEA may serve as a reliable landmark for initial position of the femoral component. Surgeons should be aware that there are outliers in this study in up to 17% of the measurements, which potentially could result in deviations of femoral component rotation.

Cite this article: *Bone Jt Open* 2024;5(12):1067–1071.

## Introduction

The rotational alignment of the femoral component in total knee arthroplasty (TKA) is essential for correct patellar tracking. Malpositioning may cause pain, impaired range of motion (ROM), and subluxation or dislocation of the patella.^1-3^ The surgical transepicondylar axis (sTEA) has been proposed as a reliable landmark to determine femoral component rotation, facilitating the appropriate balancing of both the extension and flexion gap.^4-6^ However, when using the sTEA, both the medial and lateral epicondyle need to be well preserved.

In revision TKA, severe bone loss of the distal femur may lack proper orientation for placing the femoral revision component or even complete distal femur components correctly. Such severe bone loss may occur in periprosthetic fracture, as well as in septic and aseptic periprosthetic loosening. The number of these cases is increasing and poses a challenge in achieving accurate alignment of the femoral component.^7-10^ The anterior femoral cortex has been shown to be 13.4° (SD 3.4°) externally rotated to the TEA; however, considerable variance (3.2° to 23.3°) exists, and this aspect of the femur may be compromised in revision knee arthroplasty.^11^ Consequently, the existence of a reliable landmark in the supracondylar region and its correlation with the surgical transepicondylar axis are the subjects of investigation in this study.

**Fig. 1:**
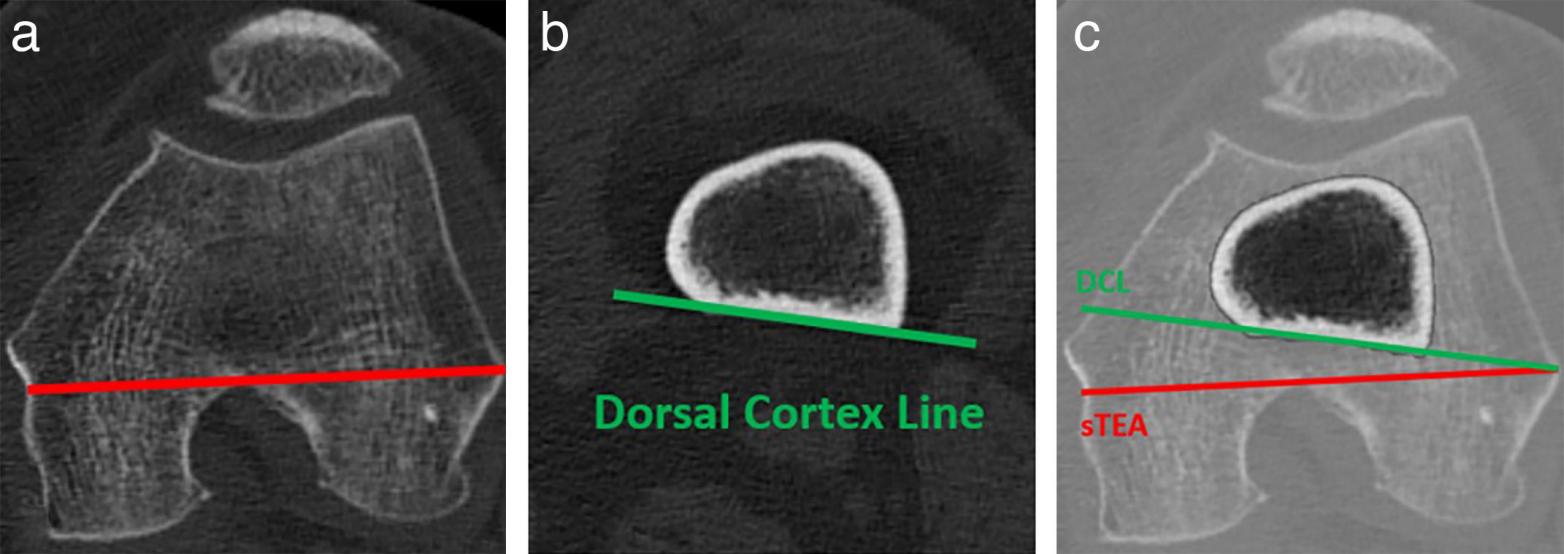
a) Surgical transepicondylar axis in a coronal CT plane; b) dorsal cortex line measured 75 mm above the joint line in coronal plane; and c) an example of overlap between surgical transepicondylar axis and dorsal cortex line.

The primary aim was to study the accuracy in measuring the dorsal cortex line (DCL) and to analyze the correlation between DCL and sTEA (Figure 1). We hypothesized that the DCL could be measured reproducibly on CT scans and that there is a correlation between DCL and sTEA.

## Methods

A total of 75 CT scans of the knee were randomly selected from patients who underwent patient-specific TKA in the database of our university hospital. These CT scans were conducted using a ten-slice CT scanner (Philips Brilliance, MRC 600 8.0 MHU, Netherlands).

This study was carried out in accordance with the World Medical Association Declaration of Helsinki.^12^ Separate ethical approval was not necessary as all data were handled anonymously within the whole data collection process.

Three experienced orthopaedic surgeons (MS, EK, MA) were assigned to measure a group of 25 CT scans twice, with a two-week interval between the measurements. In addition, each surgeon was asked to measure 25 scans from a different group once. There was no interaction between the surgeons. Consequently, each scan was measured twice by one rater and once by another rater, ensuring that no interaction effects were present.

First, the angles of the posterior condylar line (PCL) and the sTEA were measured in relation to the coronal plane. The DCL was defined in the coronal plane as a tangent to the dorsal femoral cortex located 75 mm above the joint line in the frontal plane. Subsequently, the angles between sTEA and PCL and the angles between sTEA and DCL were measured by subtracting the respective measured angles.

### Statistical analysis

A researcher (RP) who was not involved in the measurement process received the collected data and performed the analysis in a blinded fashion (RP), unaware of the purpose of the study and the raters who provided the datasets. Descriptive statistics and angulation correlations were generated for the sTEA and DCL, as well as for the distribution of measurement error for intra- and inter-rater reliability. Pearson correlation coefficient was calculated to assess the interchangeability of the sTEA and DCL, while the intraclass correlation coefficient (ICC) two-way random single measure was calculated to evaluate intra- and inter-rater reliability, respectively. SPSS Statistics v. 28.0 (IBM, USA) was used for these calculations.

## Results

All 75 randomly selected CT scans were successfully measured without any drop-outs. In the coronal plane, the PCL was 2.51° (SD 9.00°) referenced to the horizontal line, while the mean external rotation angles were 0.85° (SD 8.57°) for the sTEA and 8.26° (SD 8.28°) for the DCL. On average, an additional external rotation of 2.09° (1.47°) was observed between the sTEA and PCL. The mean external rotation of the DCL to the sTEA was a mean 9.47° (SD 3.06°), a median of 9.2° (IQR 7.45° to 11.60°), with a minimum value of 1.7° and maximum of 16.3°.

**Fig. 2:**
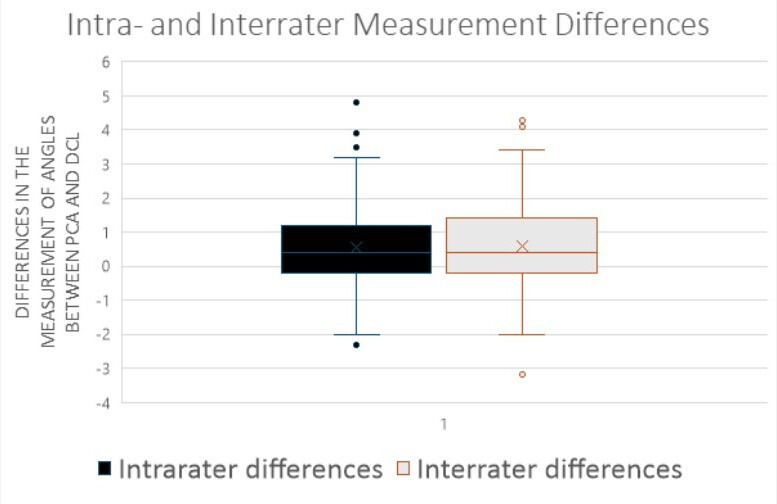
Intra- and inter-rater measurement differences for the measured angles between dorsal cortex line (DCL) and surgical transepicondylar axis (sTEA).

The measurements of the DCL demonstrated very good to excellent test-retest and inter-rater reliability coefficients, with correlation values ranging from *r* = 0.81 to *r* = 0.98, and ICC ranging from 0.80 to 0.99. The mean intra-rater measurement error for DCL was 0.59° (SD 1.22°), and between raters it was 0.58° (SD 1.30°). The IQR for the DCL measurements was between -0.10° and 1.20° of external rotation (Figure 2). Outliers were identified, with a minimum value of -2.30° (considered as an outlier, falling below 1.5-times the IQR below the first quartile) and a maximum value of 4.80°. Overall, a very good to excellent correlation between the sTEA and DCL was observed, ranging from 0.89 to 0.94. The results are summarized in Table I.

**Table I.** Main results.

Variable	PCL	sTEA	DCL
Mean α angle (SD)*	2.51 (9.00)	0.85 (8.57)	8.26 (8.28)
Mean ER to PCL (SD)		2.09 (1.47)	
Mean ER sTEA (SD)			9.47 (3.06)
Mean ICC, range			0.89 to 0.94
Mean ITE (SD)			1.09° (1.15°)

* α angle measured to horizontal line in coronal plane.

DCL, dorsal cortex line; ER, external rotation; ICC, interclass correlation; ITE, average test-retest error; PCL, posterior condylar line; sTEA, surgical transepicondylar axis.

## Discussion

The main finding of this study demonstrates good to excellent inter- and intra-rater reliability for identifying the DCL of the distal femur. This finding suggests that the DCL can serve as a reliable landmark in revision TKA when both femoral condyles are absent. The DCL was found to be approximately 10° externally rotated relative to the sTEA, with very good to excellent test-retest and inter-rater reliability coefficients observed in CT scans.

The axial alignment of TKA is not well understood,^13,14^ but the sTEA is recognized as a reliable parameter for determining femoral component rotation in both in situ and CT scan assessments.^15,16^ However, identifying the transepicondylar axis underlies a certain intra- and inter-rater variation. In a study involving six cadaver specimens, Stoeckl et al^17^ reported that, even under ideal conditions, the identification of the TEA can vary by up to 5 mm, with the identification points distributed over an area of up to 298 mm^2^. This variation could lead to potential external or internal rotation of the femoral component by up to 8° in the worst-case scenario. In the present study, the angle between the PCL and sTEA was used as a reference parameter, and the reported angles were consistent with those found in the literature.^18^ It is important to note that the current study used the sTEA, which differs from the anatomical transepicondylar axis,which is more externally rotated relative to the sTEA.^19,20^

Reliable landmarks in the supracondylar region are scarce. The distal anterior femoral cortical axis (DAFCA), as described by Sathappan et al,^11^ is one of the few bony landmarks above the epicondyles suggested for femoral component rotation in revision TKA cases involving distal femur deterioration. The DAFCA was measured approximately 60 mm from the joint line, with the limitation of being unclear when significant notching of the ventral femur from previous surgery is present. Our study introduced a suggested landmark located even higher along the dorsal cortex (75 mm above the joint line), which is independent of femoral notching. However, accessing the dorsal cortex of the distal femur requires meticulous preparation and the near absence of the distal femur, which commonly occurs in cases of severe periprosthetic fractures or malignant tumours requiring complete prosthetic reconstruction of the distal femur.^21,22^

Even though axial alignment may be normal, internal component rotation is a predominant cause of patellofemoral complications. Berger et al^23^ showed that small amounts of combined internal rotation (tibial plus femoral component) of 1° to 4° are associated with lateral tracking and patellar tilting, while moderate (3° to 8°) and severe (7° to 17°) combined component internal rotation are correlated with patellar subluxation and early patellar dislocation or late patellar prosthesis failure. The severity of the patellofemoral problem was directly proportional to the amount of internal component rotation.^23^ Similar results were reported by Matsuda et al,^24^ who found a statistically significant correlation between internal component rotation and patellar maltracking.

In revision situations with significant bone loss or destruction, the aforementioned landmarks may not be available, posing challenges in accurately determining the rotational axis of the distal femur.^25-28^ There were no outliers in the box-plot model evaluating DCL to sTEA angles. Clinically, it is noteworthy that 13 out of 75 measurements showed deviations greater than 4° from the mean of 9.47° external rotation between DCL and sTEA. According to Berger et al,^15^ this could result in complications with femoral component rotation. The minimum observed external rotation between DCL and sTEA was 1.7° and the maximum was 16.3°. Ultimately, the method allows the surgeon to identify the component rotation, which may be sufficient for patella tracking, but the tracking needs to be analyzed throughout the knee’s ROM before placing the final components.

There are limitations to our study. First, all CT scans analyzed involved patients with severe osteoarthritis who required primary TKA, and bony morphology changes in arthritic knees have been documented in the literature.^29^ Second, the study’s sample size of 75 CT scans is limited, necessitating further research to confirm our findings. Third, and most importantly, the reliability of the landmark was not investigated in a real surgical setting, meaning that identification of the landmark under surgical conditions might yield different results.

In conclusion, the DCL at 75 mm above the joint line can be measured reproducibly and is approximately 10° externally rotated to the sTEA. This may serve as a valuable landmark for determining femoral component rotation in revision TKA with significant bone loss, when the epicondyles are difficult to assess radiologically, or when the epicondyles are absent. Surgeons should be aware that there are outliers in this study in up to 17% and the trochlea design of the of the implant does not match the natural knee.

**Take home message**

- This study introduces a novel landmark for femoral component rotation in revision total knee arthroplasty with severe bone loss.

- The validity and reliability are shown in comparison to the transepicondylar axis.

- This landmark can be helpful, when the condyles are destroyed or total femur replacement is planned.

## Data Availability

The datasets generated and analyzed in the current study are not publicly available due to data protection regulations. Access to data is limited to the researchers who have obtained permission for data processing. Further inquiries can be made to the corresponding author.
